# Recognition and Risk: Ethnic Monitoring, Healthcare Access and Everyday Discrimination in Gypsy, Roma, and Traveller Communities in the UK

**DOI:** 10.1111/1467-9566.70177

**Published:** 2026-03-28

**Authors:** Győző Molnár, Peter Unwin, Rosemary (Rosa) Cisneros, Shamus McPhee, Allison Hulmes, Stacey Hodgkins

**Affiliations:** ^1^ School of Sport and Exercise Science University of Worcester Worcester UK; ^2^ School of Health and Wellbeing University of Worcester Worcester UK; ^3^ Centre for Dance Research Coventry University Coventry Pitlochry UK; ^4^ Independent Researcher UK; ^5^ School of Health and Social Care Swansea University Swansea UK; ^6^ Independent Researcher Worcester UK

## Abstract

Gypsy, Roma, and Traveller communities experience stark health inequalities in the UK, including reduced life expectancy and limited‐service access. Ethnic monitoring within the National Health Service is promoted as a tool to identify and address such inequalities, yet how these communities experience such practices remains underexplored. Drawing on 11 co‐produced focus groups with 86 self‐identified Gypsy, Roma, and Traveller participants across the UK, this article examines perceptions surrounding ethnic monitoring. Using Bourdieu's concepts of field, habitus, symbolic violence and social capital, alongside intersectionality, we reveal how disclosure of ethnicity is simultaneously desired as recognition and feared as potential stigmatisation. Participants reported identity concealment, inadequate categorisation, racism, gendered and cultural barriers and literacy and digital exclusions, while also expressing desire for visible signs of respect and cultural recognition. Ethnic monitoring emerges not as a neutral administrative practice, but as a contested site where power differentials are reproduced. Only if reframed as a practice of recognition and justice, supported by inclusive categories, cultural competence and genuine partnership with Gypsy, Roma, and Traveller organisations, can ethnic monitoring contribute to health equity.

## 1 Introduction

Gypsy, Roma, and Traveller communities are among the most marginalised minority groups in the UK and across Europe, experiencing some of the starkest health inequalities. Life expectancy for the Gypsy and Traveller communities is significantly reduced compared to the general population, with estimates suggesting that Gypsy and Traveller men live on average 10–12 years less than men in the general population, and women live 12 years less (Parry et al. 2007). This mortality gap exceeds that of any other ethnic minority group in the UK and has shown little improvement over recent decades (Office of National Statistics—ONS 2022). Maternal mortality rates are particularly alarming, with Gypsy and Traveller women experiencing rates significantly higher than the national average (Harding et al. 2004). Chronic health conditions including cardiovascular disease, respiratory illnesses and diabetes are also disproportionately prevalent, with earlier onset and poorer management compared to majority populations (Cook et al. 2013).

Mental health inequalities are equally severe. Gypsy, Roma, and Traveller communities experience higher rates of anxiety and depression, with limited access to culturally appropriate mental health services (Greenfields and Ryder 2012). Most disturbingly, suicide rates among Gypsy and Traveller communities are substantially elevated, with Irish Traveller men having suicide rates six times higher than the general population, and Gypsy and Traveller women showing similarly disproportionate rates (All Ireland Traveller Health Study Team 2010; Office for National Statistics 2022). These elevated rates reflect not only individual mental health crises but the cumulative psychological impact of systemic discrimination, social exclusion and economic marginalisation (Cemlyn et al. 2009). Despite these profound mental health needs, engagement with mental health services remains extremely low, shaped by stigma within communities, fear of child protection interventions and experiences of discrimination from healthcare providers (Unwin et al. 2025).

More broadly, Gypsy, Roma, and Traveller communities demonstrate significantly lower engagement with preventive and primary healthcare services compared to the general population (McFadden et al. 2018). Late presentation for healthcare is common, resulting in more advanced disease at diagnosis and poorer health outcomes (Condon et al. 2021). Barriers to healthcare access are multifaceted and intersecting: Experiences of direct discrimination from healthcare staff, literacy and language barriers, digital exclusion from online appointment systems, housing instability that disrupts continuity of care and profound institutional mistrust rooted in historical experiences of forced assimilation and surveillance (Cemlyn 2008; Richardson 2006; Greenfields and Rogers 2020). For many Gypsy, Roma, and Traveller individuals, the healthcare system represents not a site of care but a site of potential judgement, discrimination and state intervention (Van Cleemput et al. 2007).

Despite these well‐established disparities, structural reforms in public services, particularly the National Health Service (NHS), have struggled to provide meaningful improvements. In policy rhetoric, ethnic monitoring has been promoted as a cornerstone of the UK's equality and diversity agenda, intended to make patterns of inequality visible and enable responsive interventions (Aspinall 2020). Yet how such monitoring is experienced by Gypsy, Roma, and Traveller individuals remains underexplored.

Ethnic monitoring in healthcare refers to collecting self‐ascribed information about patients' ethnic backgrounds for equality monitoring and planning purposes. In principle, such practices aim to counteract inequalities by producing data that identify gaps in health access and outcomes (NHS England 2015). However, critics highlight its double‐edged nature: Categories may be poorly designed, experienced as intrusive or utilised in ways that reproduce rather than challenge exclusion and cultural stereotypes (Bhopal 2018; Gunaratnam 2003). Recent research demonstrates that even when ethnic monitoring occurs, misclassification rates are highest for Gypsy and Traveller groups, with almost 70% misclassified in healthcare records, resulting in systematic underestimation of severe health risks including COVID‐19 outcomes (Amele et al. 2024). For communities (e.g., Gypsy, Roma, and Travellers), who have historically faced systemic discrimination, forced assimilation and surveillance, the request to disclose ethnic identity within formal medical settings can invoke suspicion and fear (Cemlyn 2008; Richardson 2006; Unwin et al. 2025).

This article investigates how Gypsy, Roma, and Traveller communities perceive and experience ethnic monitoring practices in UK healthcare. Drawing on community member‐co‐produced focus groups (Sealey et al. 2021), we explore the duality of ethnic declaration: (1) A desire for recognition and visibility and (2) anxieties about misrecognition, stigma and potential discrimination.

This work sits at the intersection of three areas: Research on Gypsy, Roma, and Traveller health inequalities; debates on ethnic monitoring and classification and sociological theories of power and exclusion. Although scholarship on Gypsy, Roma, and Traveller health has highlighted structural barriers to equitable access (Greenfields and Rogers 2020; McFadden et al. 2018; Unwin et al. 2025), limited engagement exists with the everyday politics of ethnic categorisation in healthcare contexts. Similarly, while critical perspectives on monitoring point to risks of reification and bureaucratisation (Gunaratnam 2003), little is known about how these practices are received by Gypsy, Roma, and Traveller patients. By foregrounding participants' voices, we aim to bridge this gap.

To interpret these findings, we employ Bourdieu's (1990, 1991, 1998) conceptual triad of field, habitus and symbolic violence, supplemented with social capital. These concepts provide a lens for understanding how healthcare encounters are structured by asymmetrical power relations, how embodied dispositions of Gypsy, Roma, and Traveller individuals interact with institutional expectations, and how symbolic forms of misrecognition perpetuate exclusion. We also draw on intersectional perspectives (Crenshaw 1989; Collins 2015) to acknowledge how ethnicity, gender and class mobility intersect to shape healthcare experiences. These theoretical resources enable us to situate participants' narratives within broader structures of inequality while recognising their agency and resilience.

## 2 Theoretical Framework

Bourdieu offers a conceptual toolkit for understanding inequality and exclusion in institutional contexts. Central is the notion of field: Relatively autonomous arenas of social practice, each structured by its own logics, rules and power relations (Bourdieu and Wacquant 1992; Nairz‐Wirth et al. 2017). The healthcare system can be understood as one such field, where professionals, administrators and patients occupy different positions, and where legitimate forms of knowledge and behaviour are defined and monitored (Collyer et al. 2015; Thompson‐Lastad et al. 2025). Within this field, ethnic monitoring operates as a classificatory mechanism reflecting institutional priorities: to produce data for governance, benchmarking and policy evaluation.

Gypsy, Roma, and Traveller communities entering this field bring with them a habitus, that is, embodied dispositions, values and practical orientations shaped by their histories of mobility, community life and experiences of marginalisation (Bourdieu 1990; Jesper et al. 2008). These dispositions often clash with healthcare field expectations, which assume literate, settled and bureaucratically compliant patients (Van Cleemput et al. 2007; Unwin et al. 2025). Difficulties with digital registration systems, discomfort with written forms or gendered preferences around healthcare interactions reflect a habitus misaligned within institutional norms (House of Commons Women and Equalities Committee 2019; Office for National Statistics 2022). Such misalignment results in experiences of exclusion, frustration or resistance, which are then misrecognised as individual failings rather than structural incompatibilities.

This dynamic exemplifies what Bourdieu (1991) termed symbolic violence: The subtle taken‐for‐granted imposition of dominant norms that render minority practices invisible or devalued. Ethnic monitoring can constitute a form of symbolic violence when categories fail to reflect Gypsy, Roma, and Traveller identities (e.g., grouping ‘Gypsy/Traveller’ together or with ‘Other White’), thereby erasing difference, or when disclosure leads to discrimination. Rather than empowering patients, in such instances, monitoring may reinforce being ‘othered’.

Finally, the concept of social capital (Bourdieu 1986) highlights relational resources that communities mobilise to navigate exclusion. For many Gypsy, Roma, and Traveller communities, informal networks of kin and community play crucial roles in accessing healthcare, sharing information, interpreting bureaucratic processes or advocating in healthcare encounters. However, these forms of social capital are often devalued in institutional settings where access depends on formalised credentials and compliance with official categories and practices. Analysing ethnic monitoring through social capital underscores both the resilience of Gypsy, Roma, and Traveller networks and their structural disadvantage.

Although Bourdieu's framework illuminates structural dynamics of healthcare encounters, it has been critiqued for insufficient attention to intersecting forms of identity and oppression (Lovell 2000). We, therefore, integrate an intersectional perspective, rooted in Black feminist scholarship, formally coined by Crenshaw (1989) and further elaborated by scholars such as Collins (2015). Intersectionality has since become a wide‐spread analytical framework across disciplines, offering conceptual tools for understanding how multiple systems of power and inequality operate simultaneously and interdependently (Collins and Bilge 2016). Where Bourdieu emphasises how categories of ethnicity, gender and class structure fields of practice, intersectionality emphasises how these categories do not operate independently, but interlock to produce distinct experiences of marginalisation.

Collins and Bilge (2016) identify six core tenets of intersectionality that guide our analysis. First, social inequality is understood as produced through intersecting systems of oppression rather than single axes of difference. Second, power operates through multiple domains (structural, disciplinary, cultural and interpersonal), shaping both macro‐level policies and micro‐level healthcare encounters. Third, relationality emphasises that social categories such as ethnicity, gender, class and age gain meaning in relation to one another and cannot be analysed in isolation. Fourth, intersectional inequalities are historically specific and geographically situated, requiring attention to how local institutional practices and national policies shape experiences. Fifth, complexity acknowledges that individuals and communities occupy multiple, sometimes contradictory social positions that produce non‐additive effects. Finally, social justice orients intersectional scholarship towards identifying and challenging inequality, positioning research as a tool for transformation rather than mere description.

In health research, Bowleg (2012) argues that intersectionality moves beyond examining independent effects of social categories to understanding how intersecting identities create qualitatively distinct experiences and health outcomes. For Gypsy, Roma, and Traveller communities, this means recognising that healthcare barriers emerge not from the sum of ethnic discrimination plus gender inequality plus class disadvantage, but from the unique configurations produced by these intersecting systems. For Gypsy, Roma, and Traveller women, barriers to healthcare may be shaped simultaneously by patriarchal norms within their communities (e.g., cultural expectations around modesty and gender concordance), systemic racism in healthcare institutions and socioeconomic precarity (Condon et al. 2021). For Roma migrants, linguistic barriers intersect with xenophobia and legal precarities of migration status (Sarafian et al. 2024).

Intersectionality, thus, complements Bourdieu's concepts by foregrounding the multiplicity of oppressions converging in healthcare experiences while recognising how these are mediated through the field's logics and symbolic violence. Where Bourdieu illuminates how healthcare operates as a structured field of power relations, intersectionality ensures we attend to how individuals are differently positioned within that field based on their location at multiple intersecting axes of inequality. Together, these frameworks enable us to analyse both the structural positioning of Gypsy, Roma, and Traveller communities within healthcare systems and the heterogeneous experiences within these communities shaped by gender, age, migration history, language, literacy and socioeconomic status.

## 3 Methods

This study adopted a qualitative design to explore how Gypsy, Roma, and Traveller communities experience and understand ethnic monitoring in healthcare settings. Qualitative methods were chosen to capture lived experiences, narratives and meanings (Braun and Clarke 2021). The study was co‐produced with Gypsy, Roma, and Traveller community members and user‐led voluntary organisation partners throughout all stages of the research process. Community members with lived experience were involved in the initial research design, including formulating research questions that reflected community priorities rather than solely academic interests. The focus group topic guide was co‐developed collaboratively between academic researchers and community partners to ensure questions were culturally appropriate and addressed issues of genuine concern to Gypsy, Roma, and Traveller communities. Following data collection, community co‐researchers participated in reviewing and discussing the preliminary findings and themes identified through the qualitative analysis, ensuring interpretations remained grounded in community perspectives and experiences.

We conducted 11 focus groups with 86 participants (76 women and 10 men) who self‐identified as members of Gypsy, Roma, and Traveller communities: two in Northern Ireland, two in Wales, two in Scotland and five in England. Focus group participants comprised diverse backgrounds across the four UK nations. In England, participants included Roma, Gypsies, and Travellers; in Scotland, Scottish Gypsy Travellers and Roma; in Wales, Welsh Gypsies and Irish Travellers and in Northern Ireland, Roma, and Irish Travellers. Ages ranged from 18 to 67. The significant majority of participants were women, reflecting both the gendered nature of healthcare engagement within these communities and women's roles as primary health decision‐makers for families. Attendees were remunerated for their time in accordance with best inclusion practice (National Institute for Health Research 2024). Recruitment was facilitated by established community gatekeepers and organisations providing healthcare advocacy, education and welfare support. Partner organisations comprised: Lincolnshire Traveller Initiative, Leeds Gypsy and Traveller Exchange (Leeds GATE), Gypsy and Traveller Empowerment Herts (GATE Herts), York Travellers Trust, Sheffield Roma, Rotherham Roma, Pitlochry Scottish Gypsy Travellers, Glasgow Romano Lav, Travelling Ahead Wales and Armagh Traveller Initiative.

Focus groups were conducted between May and October 2024 at the premises of the partner charities supporting the project. Each focus group lasted approximately 90 minutes, though the research team spent considerable additional time before and after the formal interviews building relationships with staff and participants. This extended engagement was crucial for building trust and demonstrating commitment to community engagement. Refreshments were provided during all sessions, funded through the project budget. Focus groups were facilitated by two research team members: one academic researcher and one Gypsy, Roma, or Traveller community co‐researcher, to ensure cultural sensitivity and build trust. Researchers were introduced by charity staff members, after which the research team introduced the project and invited questions before commencing the discussion. A semi‐structured topic guide covered: Experiences of being asked about ethnicity in healthcare settings; perceptions of ethnic monitoring categories; experiences of discrimination, trust or fear linked to disclosure; perceived benefits or risks of declaring ethnicity and suggestions for improving ethnic monitoring and healthcare access. Discussion began with a grand tour introductory question before moving to more focused questions (Leech 2002). All sessions were audio‐recorded with consent, transcribed verbatim using OtterAI and anonymised.

Transcripts were reviewed by research team members and thematically analysed following Braun and Clarke's (2021) six‐step approach. Two researchers independently coded the data, generating initial codes inductively to capture recurrent ideas in participants' accounts. The two researchers then met to discuss emerging themes and reach consensus on the thematic framework. In cases of disagreement about coding or thematic organisation, a third researcher was brought into the discussion to adjudicate and facilitate resolution. Codes and preliminary themes were managed manually using Word documents. These inductively derived themes relating to recognition, mistrust, discrimination and barriers to inclusion were then presented to community co‐researchers and the broader research team for discussion. Following this collaborative review, the research team agreed on the final thematic structure. A second layer of analysis then applied a deductive approach, using Bourdieu's concepts of field, habitus, symbolic violence and social capital as sensitising concepts, to interpret the patterns identified in the data and situate individual narratives within wider structures of inequality. Intersectionality informed our analysis by attending to how ethnicity intersected with gender, class and other social positions in shaping participants' healthcare experiences, ensuring we recognised the multiple overlapping forms of marginalisation described by participants rather than treating Gypsy, Roma, and Traveller identity as a singular homogenous category.

Ethical approval was granted by University of Worcester. Attention was paid to informed consent, anonymity and the potential for discussing sensitive experiences of discrimination to cause distress. The research team built on platforms of established trust with Gypsy, Roma, and Traveller organisations from previous partnerships. Participants were reminded of their right to withdraw at any time until analysis began. Gypsy, Roma, and Traveller co‐researchers were involved in designing consent forms and information sheets to ensure accessibility. The research team was acutely aware of the apprehension many Gypsy, Roma, and Traveller communities hold regarding research ‘done to them’ rather than ‘with them’, and that many research reports had not led to tangible improvements in life experiences and life chances. In fact, health, educational and social care outcomes for Gypsies, Roma, and Travellers have worsened (House of Commons Women and Equalities Committee 2019; Greenfields and Rogers 2020; Office for National Statistics 2022; Unwin et al. 2023). The team was frank in recruitment and initial engagement that, although communities' views about ethnic data collection would represent new knowledge, assurances could not be given that much‐needed system changes would follow from the research findings. We used AI for locating relevant sources and formatting the reference list.

Several methodological limitations warrant acknowledgement. First, the significant gender imbalance in our sample (88% women) limits our understanding of how Gypsy, Roma, and Traveller men experience ethnic monitoring and navigate healthcare systems. The near absence of men's voices means we cannot fully capture gendered dimensions of healthcare experiences across the communities. Second, language barriers posed challenges in some focus groups, particularly with Roma participants, necessitating the use of translators. Although translators facilitated participation, translation inevitably involves interpretation and may have affected the nuance and depth of some contributions. Third, the focus group format itself presented limitations. In several sessions, participants were caring for children and babies during discussions, creating distractions that may have limited engagement with sensitive topics. Additionally, the group setting may have inhibited discussion of particularly personal or stigmatising experiences that might have been shared more readily in individual interviews. Finally, while our sample spanned all four UK nations and included diverse Gypsy, Roma, and Traveller backgrounds, recruitment through established community organisations may have reached more engaged community members with existing connections to support services, potentially missing the perspectives of more marginalised or isolated individuals within these communities (see Table 1).

**TABLE 1:** Demographics of focus group participants.

Focus group	Gender	Age range	*N*
1. English gypsies	Women 7	18–55	7
2. Roma	Women 10	18–50	10
3. Roma	Women 9/men 2	20–65	11
4. Irish travellers	Women 7/men 2	25–55	9
5. English gypsies	Women 8/men 2	24–65	10
6. Irish travellers	Women 9/men 2	22–67	11
7 English gypsies.	Women 9/men 1	25–65	10
8. Welsh Romany gypsies	Women 3	18–45	3
9. Irish travellers	Women 3	20–55	3
10. Scottish gypsy travellers	Women 5/men 1	50–65	6
11. Roma	Women 6	22–60	6

## 4 Findings

Our findings are presented thematically across four domains, drawing on 86 participants' experiences across 11 focus groups (FG). Quotations are anonymised by focus group number.

### 4.1 Recognition, Misrecognition and the Politics of Categorisation

Ethnic monitoring operates as a classificatory technology imbued with symbolic power. For Bourdieu (1991), the authority to name and categorise represents a fundamental form of symbolic violence: those who control classification systems determine which identities are recognized as legitimate and which are rendered invisible or collapsed into residual categories. From an intersectional perspective, classificatory systems privilege some social positions while systematically disadvantaging others, with majority ethnic distinctions carefully maintained, whereas minority groups are homogenised or erased (Collins and Bilge 2016). For Gypsy, Roma, and Traveller communities, ethnic monitoring in healthcare emerges as a contested site where the desire for recognition collides with fear of discrimination, and where bureaucratic categories enact misrecognition by failing to reflect lived identities.

Participants articulated profound ambivalence about ethnic disclosure. For some, declaring ethnicity represented an assertion of identity and a refusal of historical erasure:

I always tick Gypsy or Traveller if it is there. Why should I hide who I am? It is part of me, part of my family.

(FG1)

Such declarations function as claims to symbolic capital, that is, the right to be recognised as legitimate subjects within the healthcare field. However, for many others, disclosure invoked expectations of discrimination rooted in accumulated experience:

If you put Roma on the form, you are treated different straight away. The moment they see it, their whole attitude changes. It is safer to just put ‘White Other’ and not bring trouble to yourself.

(FG2)

The phrase ‘not bring trouble’ reveals discrimination as anticipated inevitability rather than possibility. This reflects what Bourdieu (1990) terms the ‘practical sense’ embedded in habitus: pre‐reflective understanding of field rules developed through repeated exposure. Concealment does not represents paranoia but practical knowledge transmitted intergenerationally:

My Mammy said never let on to anyone that you are a Gypsy. Only trouble will follow.

(FG5)

This strategic concealment constitutes a form of social capital mobilised for protection, with disclosure strategies calibrated situationally:

Sometimes I tick it, sometimes I do not. It depends on the situation, who is asking, what I need from them. If I really need that appointment or that referral, I might not risk it.

(FG9)

The participant has developed ‘disclosure literacy’, that is, the capacity to assess contexts and make rapid calculations about risks and benefits. An intersectional lens illuminates how age and generation intersect with ethnicity to shape disclosure strategies differently. Whereas older generations incorporated concealment as survival strategy forged through decades of discrimination, younger participants may seek visibility, reflecting changing social contexts and minority rights movements. One younger participant expressed the following:

My mum always says don't tell them you are Traveller… but I am tired of hiding. I want them to know who we are… But then when I do say it, I see that look in their eyes and I wonder if she is right.

(FG6)

This generational divergence operates alongside other intersecting positions: gender, migratory experience and employment status all influence the relative risks of ethnic visibility.

The inadequacy of ethnic categories emerged as acute symbolic violence. Roma participants expressed frustration:

They put Gypsy and Traveller on forms, but never Roma. It is like we don't exist. They just throw us in with Eastern European or White Other. But we are not just another Eastern European group. We are Roma.

(FG3)

When bureaucratic categories render Roma identity invisible, they enact epistemic violence, that is, denial of validity as a distinct identity worthy of institutional recognition (Sarafian et al. 2024). Another Roma participant elaborated:

When there is no Roma option, I have to decide: do I tick White even though I don't feel white? Do I tick Other and become invisible? Or do I tick Gypsy or Traveller even though I am not? None of these choices feel right.

(FG3)

Each available option represents a form of violence: claiming whiteness erases ethnicity; ‘Other’ renders one a residual category and ‘Gypsy or Traveller’ misrepresents identity. Irish Travellers similarly objected to homogenisation:

We aren't the same as Roma or English Gypsies. We are Irish Travellers with our own traditions, our own Cant, our own history. But the system doesn't see that.

(FG7)

From an intersectional perspective, this differential recognition reveals how power operates through classificatory systems. Distinctions among white British populations are carefully maintained, while minority groups like Roma are collapsed or erased. This reflects intersecting hierarchies: not only ethnic majority/minority distinctions, but citizenship status, migration history and perceived legitimacy as service claimants. The categorical system becomes a technology of power determining whose identities warrant institutional recognition and whose can be administratively erased. Homogenising diverse populations under broad categories precludes development of interventions responsive to distinct needs shaped by different migration histories, linguistic backgrounds, and experiences of discrimination. Roma communities face barriers related to recent migration and language, differing from multigenerational Gypsy or Traveller populations. Collapsing these groups erases intersecting dimensions of inequality and renders service provision not merely ineffective but potentially harmful.

Participants demonstrated sophisticated understanding of how categorical violence translates into material exclusion:

If the data doesn't show how many Roma are using services, how can they plan services for Roma? If we are all just lumped in as Other, we are invisible. And when you are invisible, your needs don't get met.

(FG9)

Categorical erasure produces data invisibility, leading to policy neglect and service inadequacy, a self‐perpetuating cycle of exclusion. Intersectionality directs attention to the need for tailored group‐specific solutions that recognise how different configurations of migration, language, settlement patterns and discrimination experiences shape distinct health needs.

The duality participants described illustrates the ‘double bind’ of ethnic monitoring for Gypsy, Roma, and Traveller communities. Invisibility perpetuates exclusion from data, policy attention and resources. Yet visibility risks exposure to discrimination and surveillance. As Goffman (1963) argued, stigmatised groups face constant calculations about disclosure management, weighing authenticity's benefits against exposure's costs. In the healthcare field, where Gypsy, Roma, and Traveller patients occupy structurally disadvantaged positions with limited capital, these calculations carry weight. The decision to ‘tick or not tick’ is never merely administrative; it represents strategic negotiation of power relations where stakes include access to care, treatment quality and psychological safety.

### 4.2 Discrimination, Mistrust, and the Surveillance Field

Healthcare operates as a Bourdieusian field structured by hierarchies, rules and differential distributions of capital (Bourdieu and Wacquant 1992). Professionals occupy dominant positions endowed with cultural and symbolic capital, whereas Gypsy, Roma, and Traveller patients enter with limited institutional capital and stigmatised identities. An intersectional analysis reveals how multiple axes of power converge within this field: Ethnicity intersects with socioeconomic position, educational capital and perceived legitimacy as claimants to shape whose knowledge is valued and whose concerns warrant institutional response (Collins and Bilge 2016). The result is encounters marked by symbolic violence, where discrimination operates not only through overt prejudice, but through everyday practices that naturalise exclusion and mark Gypsy, Roma, and Traveller bodies as out of bounds.

Participants described routine experiences of discrimination that shaped their willingness to engage with health services:

I've had many unpleasant experiences, so I probably wouldn't go to the GP, and I think other people think the same thing. Well, when we go, we are not treated like normal people, so we're not going.

(FG4)

The phrase ‘not treated like normal people’ captures fundamental denial of equal status and dignity, not occasional poor service but systematic othering. One participant recounted how discrimination operates through casual dehumanisation:

I took my mom into the doctor's surgery… So, she [receptionist] gave me the form, and she got down behind the table and said, ‘It's one of them Gypsies off of the site’.

(FG8)

That such comments occur within earshot reveals how normalised prejudice has become within some healthcare settings. The institutional authority figure enacts exclusion through language that denies patients' full humanity, exemplifying symbolic violence made palpable through everyday interaction.

More disturbingly, discriminatory attitudes persist during the most vulnerable moments:

I wouldn't want the NHS to know that I was a Gypsy. No, because the kind of comments I heard from some of the nurses, and the way they treated my relative when she was dying was horrendous, making comments like ‘Who do all these Gypsies think they are?’

(FG10)

That such attitudes surface during end‐of‐life care reveals the depth of institutional racism within some healthcare contexts. The rhetorical question positions Gypsy, Roma, and Traveller communities as presumptuous for expecting equal treatment, reflecting broader structures of symbolic violence that deny legitimacy to minorities' claims for dignity and care. These experiences create lasting trauma and fuel profound mistrust of healthcare institutions.

Power operates not only through overt discrimination but through the systematic dismissal of patient voices. When participants articulated critiques of classificatory practices or reported discriminatory treatment, their feedback rarely translated into institutional change:

I have told receptionists, told nurses, told doctors: the categories are wrong, ‘Roma’ needs to be separate. But nothing changes. They just say that is the form, that is the system. Nobody seems to have the power to change it, or maybe nobody cares enough to try.

(FG3)

Another participant elaborated:

We've done awareness raising sessions with health professionals, where we've explored good and bad practice. There's many of these meetings, and yet every time, nothing changes… You tell them the same things over and over again and they never take them any further.

(FG11)

This reflects the hierarchical nature of the healthcare field: Patients occupy dominated positions with limited authority to shape institutional practices, whereas decision‐makers who could revise systems are insulated from feedback. The observation that ‘You tell them the same things over and over again and they never take them any further’ exemplifies epistemic injustice and that knowledge claims from marginalised positions are systematically discredited. This dismissal is compounded for those experiencing additional marginalisations based on gender, literacy levels or immigration status.

Mistrust was particularly acute concerning potential data sharing with authorities, especially child protection services. This fear reflects not paranoia but historical patterns of disproportionate interventions in the Gypsy, Roma, and Traveller communities, where child removal has functioned as a tool of assimilation and cultural destruction (Cemlyn 2008). One participant articulated the profound dilemma:

I do worry about having our children taken away. If you go to A&E with a child you have to stay, even if the wait is really long because you're scared to leave, or they'll have the Social Services on you.

(FG9)

Fear of surveillance shapes healthcare behaviour in extreme ways and, consequently, parents endure unreasonable waits rather than risk being reported for ‘inadequate’ parenting. This fear operates as social control, demanding compliance through threat of family separation. The long‐term consequences are devastating:

I agree health [NHS] should know who we are, but it took me nearly 20 years to get help for my mental health because I was so frightened that social workers would come and take my babies if I said I had depression.

(FG1)

A woman suffered untreated mental illness for two decades because fear of child removal outweighed her need for healthcare. The participant's framing (‘I agree health services should know who we are, but…’) demonstrates the ambivalence of ethnic monitoring: recognition that visibility could enable better services, tempered by well‐founded fears of punitive consequences.

Intersectionality emphasises that social context fundamentally shapes how inequalities are experienced and reproduced (Collins and Bilge 2016). Ethnic monitoring practices vary across healthcare settings, with hospitals, GP surgeries and health visitors each using different classificatory schemes. A Roma woman seeking maternity care may face different categorical options, levels of cultural competence and discrimination risks than when accessing mental health services or emergency care. These contextual variations mean ethnic disclosure carries different risks and benefits across settings, requiring constant recalibration of strategies. The intersection of ethnicity with other positions produces distinct surveillance experiences and vulnerabilities that cannot be understood through ethnicity alone. Social context is, thus, not merely backdrop to inequality, but actively constitutes how intersecting marginalisations are enacted and experienced. Healthcare functions not merely as a site of care but as a site of potential surveillance, with ethnic monitoring implicated in these dynamics of control.

### 4.3 Structural Barriers: Digital Exclusion and Cultural Capital

The digitalisation of healthcare systems represents what Bourdieu (1986) would identify as a mechanism requiring specific forms of cultural and economic capital, that is, resources unequally distributed across social groups. Digital literacy, internet access and familiarity with online platforms function as gatekeeping resources that increasingly determine access to essential services. For Gypsy, Roma, and Traveller communities with lower rates of formal literacy and limited digital infrastructure access, this transformation erects profound barriers. Intersectionality reveals how ethnicity, age, literacy levels and economic resources converge to create compounded exclusion that cannot be understood through any single axis alone, with digital healthcare effectively functioning as a mechanism widening existing health inequalities for those positioned at multiple intersecting sites of marginalisation (Bowleg 2012).

Participants described how digital‐first systems exclude older community members:

My dad and mum, they're diabetics, and there's a new system where you've got to go online to order a repeat prescription, and they've made them aware that they can't do that because they have no internet. They don't even use a debit card, right?

(FG10)

Despite explicit notification of barriers, the system persists with digital‐only access, effectively denying diabetic patients the ability to obtain essential medication. The phrase ‘don't even use a debit card’ acknowledges generational dimensions to digital exclusion, yet highlights institutional inflexibility that prioritises efficiency over equity, demonstrating how the absence of requisite capital translates directly into service exclusion.

The consequences extend beyond prescription management to appointment systems, creating cascading effects:

They don't know how to do all that computer stuff, so they're missing more appointments than what they're gaining. If you miss it twice, you're out with a dentist.

(FG9)

This testimony reveals compounding effects of digital and literacy barriers across generations. Older parents lack digital skills, younger family members may lack literacy skills and communication systems rely on email, online portals and written letters that neither generation can always navigate. The result is missed appointments that are then punished through service exclusion rather than recognised as symptomatic of inaccessible communication systems. That dental services exclude patients for missed appointments without considering whether systems are accessible represents symbolic violence: Structural barriers are misrecognised as individual irresponsibility.

The burden of navigating digital systems falls disproportionately on younger digitally literate family members, mobilising intergenerational social capital to compensate for institutional inflexibility. Yet this places unsustainable demands on families and proves unreliable as younger members juggle their own responsibilities. Social capital that functions effectively within community contexts (kinship networks providing mutual support) struggles to convert into institutional recognition within healthcare systems privileging formal credentials and digital literacies. The shift to digital‐first or digital‐only healthcare represents structural violence that systematically excludes communities with lower rates of digital and literacy capital, perpetuating health inequalities under the guise of modernisation. For communities already experiencing ethnic discrimination, inadequate categorical recognition and surveillance anxieties, digital transformation adds another layer of exclusion, demonstrating how intersecting systems of inequality compound to create multiple simultaneous barriers to healthcare access.

### 4.4 Gendered Habitus and Intersecting Vulnerabilities

Habitus is differentiated not only by ethnicity but also by gender, producing distinct embodied dispositions around appropriate behaviour and healthcare interactions (Bourdieu 1990). For Gypsy, Roma, and Traveller women, cultural expectations around modesty and gender concordance frequently clash with institutional healthcare arrangements that prioritise efficiency and specialist allocation over cultural accommodation. Intersectionality illuminates how ethnicity and gender converge to create distinctive patterns of healthcare exclusion. Women face not only cultural barriers around gender concordance and modesty, which shape all women's healthcare seeking within their communities but also ethnic discrimination, marking all Gypsy, Roma, and Traveller patients as Other within healthcare settings (Collins and Bilge 2016). Simultaneously, gendered expectations position women as primary caregivers whose own health needs are subordinated to family care responsibilities. These intersecting constraints produce systematic delays in healthcare seeking that are then misrecognised by healthcare professionals as individual irresponsibility rather than as predictable outcomes of intersecting structural inequalities.

One participant articulated multiple dimensions of distress produced when healthcare systems cannot accommodate cultural gender norms:

Health people need to know more about what it means to be a Gypsy woman… You can't talk about certain things with men, you just can't do it. When I've had to see a man doctor because he was the specialist, I had no choice. I feel uncomfortable. I find it hard to talk to him about it, I find it embarrassing trying to talk to a male gynaecologist. It is not culturally allowed to talk to a man about such things. I couldn't tell me dad it was a man, I lied to his face and felt bad about it.

(FG5)

This testimony reveals multiple layers of distress: Physical discomfort and embarrassment in the clinical encounter, cultural transgression and family deception and guilt. The phrase ‘I had no choice’ captures the coercive dimension, when specialist care is required, cultural needs are overridden by organisational constraints. This represents collision between gendered habitus (embodied dispositions around appropriate behaviour for women) and healthcare field logic prioritising efficiency over cultural accommodation. Condon et al. (2021) found that cultural barriers around gender concordance were significant factors in low uptake of cancer screening among Gypsy, Roma, and Traveller women, with serious health outcome implications.

Healthcare professionals who frame these cultural needs as ‘fussiness’ or ‘awkwardness’ exemplify symbolic violence:

They think it's you just being fussy, being awkward because they said to me, ‘Well, you've got a chaperone,’ and I say: ‘I know, but I just don't want to see the man’. I try and word it like in a nice way, but I think they just see it's like ‘you're being awkward’.

(FG6)

Legitimate cultural requirements are delegitimised and reinterpreted as individual unreasonableness. The presence of a chaperone is presented as sufficient accommodation, yet this misses the fundamental point that cultural norms preclude discussion of intimate health matters with men, regardless of who else is present. The participant's efforts to ‘word it like in a nice way’ reveal emotional labour of negotiating cultural needs within a system framing those needs as illegitimate demands. This interaction reinforces healthcare field power asymmetry, where professionals define what constitutes reasonable accommodation and patients must justify cultural practices differing from institutional norms. These gendered cultural expectations extend beyond individual preferences to family and community norms:

We don't want to have [health]care from a man, imagine letting a man try to lay a hand on our dear old mother. It just wouldn't be allowed to happen.

(FG11)

The phrase ‘wouldn't be allowed to happen’ indicates the strength of these norms and collective enforcement mechanisms supporting them. These are deeply embodied dispositions that cannot simply be overridden without violating fundamental aspects of identity and cultural belonging.

Gendered expectations around caregiving produced additional healthcare challenges. One participant described how caring responsibilities intersect with health‐seeking behaviour:

As a Traveller woman, you're expected to look after everyone: the kids, your husband, your parents, maybe your in‐laws too. Your own health comes last. So, by the time you get to the doctor, things have got really bad. But then they judge you for leaving it so long, for not coming in sooner. They don't understand the pressures you're under, that you don't have time to be sick.

(FG10)

The phrase ‘you don't have time to be sick’ captures how structural constraints and gendered care expectations, are embodied as dispositions of self‐neglect. Women's health needs are systematically deprioritised within family structures where they bear primary caring responsibilities for multiple generations. Yet when this manifests as delayed presentation, it is interpreted through a deficit lens rather than recognised as the product of gendered expectations. An intersectional perspective reveals how gendered habitus, combined with experiences of ethnic discrimination and systemic barriers around digital access and literacy, produces particularly low engagement among Gypsy, Roma, and Traveller women with preventive healthcare services, contributing to poor health outcomes including later‐stage cancer diagnoses and untreated chronic conditions (Office for National Statistics 2022). The intersection of ethnicity and gender creates distinctive patterns of healthcare exclusion requiring interventions responsive to both dimensions of lived experience.

## 5 Discussion

This study explored how Gypsy, Roma, and Traveller communities experience and interpret ethnic monitoring in healthcare. The findings reveal dynamics of recognition, mistrust, discrimination and ambivalence that not only expose immediate barriers to access, but also illustrate how broader configurations of field, habitus, symbolic violence and capital shape the lived realities of Gypsy, Roma, and Traveller people within the UK healthcare system. This research makes three key sociological contributions: Advancing theoretical understanding of how classificatory systems reproduce inequality; demonstrating the utility of combining Bourdieusian and intersectional frameworks for analysing minority health experiences and revealing the mechanisms through which well‐intentioned equity interventions can paradoxically perpetuate the exclusions they seek to remedy.

Bourdieu's concept of symbolic violence illuminates how practices presented as neutral or technical, such as ethnic monitoring, can perpetuate cultural domination. Monitoring is framed as an instrument for equality, yet participants' accounts show it can be experienced as stigmatising. The homogenisation of distinct groups into a single ‘Gypsy or Traveller’ category exemplifies how bureaucratic classifications impose dominant logics that erase lived distinctions. For Bourdieu (1991), symbolic power lies in the authority to classify and name. When categories do not reflect self‐understandings, ethnic monitoring enacts misrecognition by rendering some identities invisible and others hyper‐visible. Therefore, rather than challenging inequality, the classificatory system risks reproducing disenfranchisement by reinforcing the marginal status of Gypsy, Roma, and Traveller communities. This finding extends sociological scholarship on categorisation and governmentality by demonstrating how technologies of equality monitoring can simultaneously function as technologies of erasure, revealing the paradoxical nature of recognition politics within bureaucratic systems.

Participants' reluctance to disclose ethnicity reflects not only strategic calculation but also embodied histories of discrimination. For Bourdieu (1990), habitus comprises dispositions shaped by past conditions. In this case, experiences of prejudice, surveillance and marginalisation have cultivated expectations of discrimination that inform present‐day practices. Concealment becomes the ‘practical sense’ of survival. The preference to tick ‘White Other’ rather than ‘Gypsy’ or ‘Traveller’ illustrates how habitus incorporates learnt strategies of self‐protection. Such practices are not reducible to individual mistrust; they reflect collective histories sedimented in dispositions and carried into healthcare settings. This reveals how institutional memory operates at the level of embodied practice: communities develop protective habitus not through formal instruction, but through intergenerational transmission of survival strategies, demonstrating the durability of symbolic violence across eras.

The study also highlights the role of social capital in navigating health systems. Participants often relied on kinship networks or voluntary organisations to overcome literacy barriers, interpret forms or advocate for fair treatment. For Bourdieu (1986), social capital refers to resources accessible through networks of relationships. In Gypsy, Roma, and Traveller communities, bonding capital within families and bridging capital (Putnam 2000) via trusted organisations are critical for accessing healthcare. Yet these resources are undervalued in institutional contexts where bureaucratic compliance and formal credentials dominate. Although social capital functions effectively within community contexts, it struggles to translate into institutional recognition within healthcare systems that privilege formal credentials and bureaucratic literacies.

Healthcare can be conceptualised as a field structured by its own rules, hierarchies and forms of capital (Bourdieu and Wacquant 1992). Professionals occupy dominant positions endowed with cultural and symbolic capital, whereas Gypsy, Roma, and Traveller patients enter the field with limited institutional capital and stigmatised identities. The result is encounters marked by tension and misrecognition. Participants described how boundaries of belonging are policed within the healthcare field. Such statements reinforce outsider status and signal that Gypsy, Roma, and Traveller patients do not ‘fit’ expected norms. These moments exemplify how symbolic violence operates through everyday practices to naturalise exclusion. The micro‐level interactions documented in this study reveal the mechanisms through which field boundaries are maintained and legitimated, contributing to sociological understanding of how institutional exclusion operates not only through formal policies, but through mundane communicative practices that mark certain bodies as out of place.

Although Bourdieu's framework foregrounds structural positioning, it is important to recognise that habitus is differentiated by gender, class and other social divisions. For Gypsy, Roma, and Traveller women, reluctance to consult male doctors for intimate care reflects a gendered habitus shaped by both community norms and broader structures of inequality. Similarly, limited literacy and digital exclusion intersect with economic marginalisation to compound barriers. These findings show how habitus is not monolithic but intersectional, varying within communities while still structured by overarching conditions of marginalisation. By integrating intersectionality with Bourdieusian analysis, this study demonstrates the analytical power of combining frameworks may seen as incompatible: Where Bourdieu illuminates structural positioning within fields, intersectionality reveals the heterogeneous experiences within structurally similar positions.

A central theme was duality of ethnic categorisation. Participants feared ethnic disclosure due to possible discrimination yet also sought recognition and respect. Calls for visible inclusion, such as displaying the Roma flag, demonstrate a demand for symbolic capital within healthcare spaces. Bourdieu (1991) reminds us that symbolic capital is not only about prestige but also about being recognised as legitimate. Ethnic monitoring risks reproducing symbolic violence when it reduces recognition to bureaucratic tick‐boxes. Yet, if reconfigured in dialogue with communities, it could function as a form of symbolic capital, affirming identities and building trust. This finding has important implications for understanding recognition politics in contemporary institutions: The demand is not simply for statistical visibility but for meaningful acknowledgement that confers legitimacy and respect. Ethnic monitoring frameworks must therefore recognise diversity within Gypsy, Roma, and Traveller communities, including distinct categories rather than collapsing groups into homogenised labels. The inconsistency of categories across healthcare settings, where hospitals, GP surgeries and community services each use different classificatory schemes, further undermines legitimacy and produces incoherent data that defeats monitoring's stated purpose. Standardised, inclusive categorisation is essential if ethnic monitoring is to move beyond symbolic violence towards genuine recognition.

Through Bourdieu's concepts of field, habitus, symbolic violence and capital, alongside intersectional analysis, the findings reveal ethnic monitoring as a practice deeply embedded in power differentials. It is not merely an administrative mechanism, but a site where historical exclusions are reproduced and where recognition and misrecognition collide. For Gypsy, Roma, and Traveller communities, engaging with the healthcare field entails negotiating embodied dispositions of mistrust, deploying social capital to overcome barriers and navigating the symbolic violence of inadequate classifications. Simultaneously, the desire for recognition points to the possibility of transforming ethnic monitoring into a practice that affirms dignity and contributes to healthcare equity. Realising this transformation requires addressing the structural conditions that shape healthcare encounters. Healthcare providers must actively demonstrate inclusion through visible signs of respect, cultural symbols in clinical spaces, public statements of commitment and tailored health promotion materials, alongside substantive efforts to combat discrimination in practice. These symbolic gestures matter not as mere window‐dressing but as signals that shift the affective dimensions of the healthcare field, potentially altering patients' expectations and habitus over time.

Therefore, cultural competency training for healthcare professionals must move beyond generic diversity awareness to address the specific histories, cultural practices and health needs of Gypsy, Roma, and Traveller communities, co‐designed with community organisations and attending to gendered dimensions of care. The digital transformation of healthcare services poses acute challenges for communities with low literacy rates and limited digital access. A commitment to equity requires maintaining alternative access routes including face‐to‐face registration, paper‐based appointment systems and telephone services as permanent structural requirements for inclusive healthcare systems.

Critically, voluntary organisations serving Gypsy, Roma, and Traveller communities already play pivotal roles in mediating healthcare access, providing advocacy, interpreting bureaucratic processes and building trust between communities and institutions. Yet these organisations operate with precarious funding and limited institutional recognition. Policy should formalise and adequately resource these roles, integrating community advocates into healthcare pathways rather than relying on them as informal volunteer‐dependent supplements. Such integration would convert the existing social capital of community networks into institutionally recognised bridging capital, strengthening healthcare engagement while respecting community autonomy. The measures required to address these barriers are neither technically complex nor prohibitively expensive. What they demand is political will to prioritise equity and sustained institutional commitment to dismantling discriminatory practices. The stark health inequalities experienced by Gypsy, Roma, and Traveller communities, including significantly reduced life expectancy, elevated maternal and infant mortality and substantially higher suicide rates, represent a profound failure of health equity that ethnic monitoring, if properly implemented, could help to address.

## 6 Conclusion

This study has shown that ethnic monitoring in healthcare is not a neutral technical exercise, but a contested practice shaped by histories of discrimination, structural inequalities and cultural identities. For Gypsy, Roma, and Traveller communities, declaring ethnicity involves both risks and potential benefits, producing ambivalence that cannot be resolved through technical adjustments alone. The sociological contribution of this research lies in demonstrating how well‐intentioned equality interventions can paradoxically perpetuate the inequalities they aim to remedy when designed without meaningful community engagement. By applying Bourdieu's concepts of field, habitus, symbolic violence and social capital alongside an intersectional lens, we have shown how ethnic monitoring becomes a site of symbolic violence when it imposes categories that misrecognise lived identities and demands disclosure from communities with learnt expectations of punitive consequences. Yet the same practice, if reconfigured through genuine partnership with Gypsy, Roma, and Traveller organisations, could function as a tool of recognition and justice. Ultimately, healthcare for Gypsy, Roma, and Traveller communities must be reframed not simply as a question of access but as a matter of justice.

## Author Contributions

**Győző Molnár:** conceptualization, investigation, funding acquisition, writing – original draft, writing – review and editing, methodology, data curation, supervision, project administration, formal analysis. **Peter Unwin:** investigation, funding acquisition, writing – review and editing, project administration, writing – original draft, formal analysis. **Rosemary (Rosa) Cisneros:** investigation, funding acquisition, writing – review and editing, validation. **Shamus McPhee:** investigation, funding acquisition, writing – review and editing, validation. **Allison Hulmes:** investigation, funding acquisition, writing – original draft, writing – review and editing, validation. **Stacey Hodgkins:** investigation, funding acquisition, writing – review and editing, validation.

## Funding

This research received funding from the Understanding Patient Data.

## Conflicts of Interest

The authors declare no conflicts of interest.

## Data Availability

The data that support the findings of this study are available on request from the corresponding author. The data are not publicly available because of privacy or ethical restrictions.
